# Assessment and Rehabilitation Intervention of Feeding and Swallowing Skills in Children with Down Syndrome Using the Global Intensive Feeding Therapy (GIFT)

**DOI:** 10.3390/children11070847

**Published:** 2024-07-12

**Authors:** Silvia Franceschetti, Marco Tofani, Serena Mazzafoglia, Francesca Pizza, Eleonora Capuano, Massimiliano Raponi, Gessica Della Bella, Antonella Cerchiari

**Affiliations:** 1 Management and Diagnostic Innovations & Clinical Pathways Research Area, Neurorehabilitation and Adapted Physical Activity Day Hospital, Bambino Gesù Children’s Hospital, IRCCS, 00165 Rome, Italy; silvia.franceschetti@opbg.net (S.F.); serena.mazzafoglia@opbg.net (S.M.); francesca.pizza@opbg.net (F.P.); eleonora.capuano@opbg.net (E.C.); gessica.dellabella@opbg.net (G.D.B.); antonella.cerchiari@opbg.net (A.C.); 2 Management and Diagnostic Innovations & Clinical Pathways Research Area, Professional Development, Continuous Education and Research Service, Bambino Gesù Children’s Hospital, IRCCS, 00165 Rome, Italy; 3 Department of Life Sciences, Health and Allied Healthcare Professions, Università degli Studi “Link Campus University”, 00165 Rome, Italy; 4 Management and Diagnostic Innovations & Clinical Pathways Research Area, Medical Directorate, Bambino Gesù Children’s Hospital, IRCCS, 00165 Rome, Italy; massimiliano.raponi@opbg.net

**Keywords:** Down syndrome, dysphagia, children, speech and language therapy, rehabilitation, chewing, feeding

## Abstract

Background: Children with Down syndrome (DS) experience more difficulties with oral motor skills, including chewing, drinking, and swallowing. The present study attempts to measure the preliminary effectiveness of Global Intensive Feeding Therapy (GIFT) in DS. GIFT is a new rehabilitation program addressing the specific difficulties and needs of each child, focusing on sensory and motor oral abilities. It follows an intensive schedule comprising 15 sessions over 5 consecutive days, with 3 sessions per day. The principles of GIFT are applied with specific objectives for DS. Methods: GIFT was preliminarily implemented among 20 children diagnosed with DS. To measure the efficacy of GIFT, the Karaduman Chewing Performance Scale (KCPS), the International Dysphagia Diet Standardization Initiative (IDDSI), and the Pediatric Screening–Priority Evaluation Dysphagia (PS–PED) were used. Data were analyzed using the Wilcoxon signed-rank test before (T0) and after intervention (T1) and at one-month follow-up (T2). The effect size was also measured for specific outcomes, using Kendall’s W. Results: Our findings revealed that children with DS showed no risk of dysphagia according to the PS–PED (mean score 2.80). Furthermore, statistically significant improvements in chewing performance were observed, as measured by the KCPS (*p* < 0.01), as well as in texture acceptance and modification, as measured by the IDDSI post-intervention (*p* < 0.01). For both the KCPS and IDDSI, a large effect size was found (Kendall’s W value > 0.8). Parents/caregivers continued using GIFT at home, and this allowed for a positive outcome at the one-month follow-up. Conclusions: GIFT proved to be effective in the rehabilitation of feeding and swallowing disorders in children with DS, as well as for food acceptance.

## 1. Introduction

Down syndrome (DS) is a genetic disorder caused by the partial or complete presence of an extra copy of chromosome 21 [1]. In Europe, between 2011 and 2015, it is estimated that 8031 annual live births of children had a diagnosis of DS, with a prevalence of 10.1 per 10,000 live births [2]. Children with DS are at a heightened risk of developing various clinical comorbidities that necessitate regular medical monitoring. These conditions include congenital heart defects, thyroid abnormalities, respiratory issues, obstructive sleep apnea, dysphagia, and gastroesophageal reflux disease [3,4,5,6].

Specific anatomical and functional features are commonly observed, including nasal bone depression; a flat facial profile; a high palate; hypotonic perioral muscles; a relatively large, protruding, and hypotonic tongue; a narrowed oropharynx; inclined palpebral fissures; strabismus; and dental anomalies in both number and shape [7]. The presence of reduced mandibular development, which favors lingual protrusion, makes lip occlusion difficult and often causes dental problems such as agenesis or the presence of supernumerary teeth [1]. Bruxism is more prevalent in DS individuals than in the general pediatric population [8].

Oral feeding difficulties have frequently been included in the literature among the characteristics of the syndrome [9,10]. The effects of feeding and swallowing difficulties in children may lead to further medical problems (dehydration, malnutrition, growth retardation, reduction in muscle strength, weakening of the immune system), worsening the state of health and reducing potential rehabilitation [11], while increasing the hospitalization rate in the first 3 years of life of the DS population [9,12,13].

Some studies have demonstrated delayed food acceptance in DS children, with poorly coordinated movement in food management from oral to pharyngeal, difficulty in managing solid consistency, and overall reduced jaw control [14,15,16]. Multiple factors contribute to impaired masticatory performance, including the extent of tooth contact; occlusal force; and the coordination of lip, cheek, tongue, and jaw movements. Children with DS often exhibit underdeveloped midfaces and malocclusion. The absence or weakness of tongue lateralization movements can hinder the transportation of food under the teeth and the formation of a cohesive food bolus within the oral cavity [17].

The characteristics described above lead to a delay in the development of oral motor skills in feeding and swallowing. In the literature, there is little evidence regarding the treatment of oral and sensory motor functions of feeding and swallowing in children with DS. The objective of this research is to measure the effectiveness of Global Intensive Feeding Therapy (GIFT) [18] in a group of children with DS.

## 2. Materials and Methods

A pilot study was carried out during the period from September 2022 to June 2023 at the Bambino Gesù Children’s Hospital Eating and Swallowing Disorders Service.

### 2.1. Participants

This study included a convenience sample of children with a diagnoses of DS who had different functional limitations concerning oral feeding abilities. To be included in the study, participants met the following inclusion criteria: age 1–18, confirmed diagnoses with genetical examination, clients of Dysphagia Unit of the Bambino Gesù Children’s Hospital IRCCS. Children with other concomitant syndromes or neurological deficits that could explain food aversion were excluded. Children with acquired or congenital deficits to the oral cavity, pharynx, or larynx were also excluded.

### 2.2. Global Intensive Feeding Therapy (GIFT)

GIFT [18] is an intensive rehabilitation program founded on the principles of neuroplasticity [19], individualized to address the unique challenges and needs of each child. Following clinical assessment, the child participated in a rigorous rehabilitation regimen consisting of 15 sessions over 5 consecutive days, with 3 sessions per day, corresponding to breakfast, morning snack, and lunch. This intensive training was conducted by a speech-language pathologist (SLP) in collaboration with the child’s parents or caregiver. This allowed them to repeat the techniques at home in order to help the child generalize the skills learned with the SLP during the intensive treatment and to develop new ones. The main goals of GIFT are (a) to reduce oral, perioral, and upper limb hypersensitivity; (b) to promote the development of adequate chewing and swallowing skills; (c) to expand the food repertoire (in terms of quantity and variety); and (d) to reduce dysfunctional mealtime behavior.

In this study, GIFT has focused on (a) stabilizing the correct postural alignment; (b) systematic desensitization and gradual exposure; (c) food texture adaptation; (d) chewing and swallowing abilities; (e) reducing and eliminating the use of infant devices (pacifier and bottle); (f) reducing inappropriate mealtime behaviors; and (g) educating and training caregivers. The GIFT protocol can be summarized as follows:

***Stabilize correct postural alignment.*** Before promoting oral sensory and motor skills, it is essential to provide the child with correct postural alignment, which includes proper alignment of the head, neck, and pelvis stabilization. Motor anomalies, which are more common in DS, are correlated with hypotonia, joint hypermobility, and ligamentous laxity. Specifically, bony anomalies of the cervical spine can produce atlanto-occipital and cervical instability. All these characteristics often lead to the development of abnormal postural control, with consequent instability and reduced performance [20,21].

**Systematic desensitization and gradual exposure.** This study centers on sensory challenges, which are crucial for food acceptance. The goal is to acclimate children to new foods by familiarizing them with various attributes such as smell, taste, shape, color, and texture. Through a structured series of sensory experiences, children will develop confidence with food. This process involves a hierarchical approach, starting with visual acceptance, then moving to interaction, smell, and touch—beginning with parts of the body furthest from the mouth and gradually advancing to the perioral area, tasting, and finally, eating the food [22,23,24]. The protocol for introducing new foods consists of the following stages: (1) ensuring the child can visually tolerate the food when it is placed directly in front of them; (2) encouraging the child to touch, pick up, and transfer the food to another plate; (3) prompting the child to pick up, smell, and then place the food on another plate; (4) having the child pick up, kiss, and transfer the food to another plate; (5) getting the child to lick the food, making tongue contact; and (6) encouraging the child to touch the food with their teeth. The amount of food given is gradually increased with chewing guidance until the child can complete the entire meal, including both main courses.

**Food texture adaptation.** The textures of the foods proposed to the child are coherent with starting the oral sensory and motor abilities of feeding and swallowing. The following rehabilitation process is recommended for children who eat only pureed foods: (a) substitution of industrial homogenized food with fresh mixed food; (b) division of the meal into two courses with a first and second course pureed; (c) gradual increase in texture (from pureed to minced food); (d) presentation of soft solid foods with the technique of guided chewing training; and (e) administration of a whole meal with the guided chewing training.

**Chewing and Swallowing Abilities.** Subsequently, a chewing training regimen is implemented to consolidate or develop the child’s oral motor abilities through guided chewing practice trough practice and repetition [25,26]. Distinguishing tongue movements from jaw movements is crucial for developing tongue lateralization and posteriorizing skills. This practice enhances muscle tone and improves the child’s ability to handle food and saliva within the oral cavity [27,28].

**Reduce and eliminate the use of infant devices** (pacifier and bottle). It is necessary for the correct development of the oro-facial structures and oral motor skills of feeding to gradually reduce, until eliminated, the use of a pacifier, bottle, straws, or bottles with rigid spouts and to introduce the use of a glass and administer pureed foods with a spoon in order to reduce dysfunctional tongue movement. The use of infant devices compromises sensory and motor oral experiences and the typical development of feeding abilities [29,30].

**Behavioral problems during mealtime.** After a first observation of behavioral issues, the child’s behavioral problems during the meal are regulated by positive and negative reinforcement, such as using devices and/or interesting toys [30].

**Caregiver’s education and training.** The integration of parents and caregivers into the rehabilitation intervention is considered a best practice in the literature for early intervention in pediatric rehabilitation [31]. Initially, caregivers observe these methods, then gain supervised hands-on experience with their child. This allows them to learn the techniques and confidently apply them at home. Caregivers receive guidance on managing meals and performing rehabilitation techniques, which are continued after the intensive training period concludes.

### 2.3. Assessment Tools and Outcome Measures

**Pediatric Screening–Priority Evaluation Dysphagia (PS–PED).** The PS–PED is a screening tool used to assess the risk of dysphagia in pediatric patients. It consists of 14 items divided into 3 main domains: medical history, health status, and feeding condition. Patients who obtain a score from 0 to 6 present a low risk of dysphagia; patients who score from 7 to 14 are at medium-high risk of dysphagia [32].

**Karaduman Chewing Performance Scale (KCPS).** The KCPS is a functional instrument that is valid, reliable, quick, and easy to use clinically for assessing chewing function in children. It evaluates chewing function with the following scoring system: 0 indicates normal chewing function; 1 indicates the child chews but has some difficulty forming a food bolus; 2 indicates the child starts to chew but cannot keep the food in the molar area; 3 indicates the child bites but cannot chew; and 4 indicates the child cannot bite or chew. As the scale had not been translated and validated in Italian, an independent translation was conducted using the English version from the validation study by the original developers [33].

**International Dysphagia Diet Standardization Initiative (IDDSI).** The IDDSI is an entity which aims to determine the number of food texture and drink thickness levels for international standardized use [34]. The IDDSI framework consists of a continuum of eight levels (0–7) in which drinks are measured from level 0 to 4, while foods are measured from level 3 to 7, as follows: level 3 liquidized/moderately thick; level 4 pureed; level 5 minced & moist; level 6 soft & bite-sized; and level 7 regular. The IDDSI ratings are intended to confirm the textural characteristics of food and liquid at the time of testing [35].

PS–PED, KCPS, and IDDSI texture classifications were administered at pre-intervention (T0), post-intervention (T1), and one-month follow-up (T2) by experienced speech and language therapists.

### 2.4. Data Analysis

Sociodemographic data and clinical characteristics were analyzed using frequency distributions, mean (SD), and median values. The KCPS, PS–PED, and IDDSI data were assessed at three time points: before treatment (T0), after treatment (T1), and during follow-up (T2). First, to measure variance in scoring, the Friedman test was used. The Friedman test compares the difference between more than two related groups, such as comparing the difference between three time points. The null hypothesis is that the distribution is the same across repeated measures. Kendall’s W coefficient was used to measure the Friedman test effect size, which uses Cohen’s interpretation guidelines of 0.1–<0.3 (small effect), 0.3–0.5 (moderate effect), and >0.5 (large effect). However, to measure where the difference is, we need to measure the post-hoc test. The Wilcoxon signed-rank test, a common nonparametric test for paired data involving pre- and post-treatment measurements from independent units of analysis, was employed to investigate the differences among median values. The significance level was set at alpha < 0.05.

## 3. Results

The study lasted for six months involving a total of 20 children (8 F and 12 M) with a mean (SD) age of 4.85 (2.43). We first used the PS–PED as a screening tool to evaluate the risk of dysphagia, and we found no risk of dysphagia (mean score 2.8); therefore, it was possible to approach GIFT without any risk of aspiration and penetration. The whole sample participated at each session during the training. Sample characteristics are summarized in Table 1.

Despite the sample showing no risk of dysphagia, we decided to report the main results of each item of the PS–PED, highlighting that the items that mainly contributed to the PS–PED score were # 4, 11, 13 and 3. Results are synthetized in Table 2.

Concerning chewing abilities, the Friedman test demonstrated statistically significant differences with a *p* < 0.001 with a large effect size (Kendall’s W value 0.89); the Wilcoxon signed-rank test and the KCPS showed statistically significant differences (*p* < 0.001) in scoring at different timings of administration. Table 3 synthetizes mean (SD) and median (IQR) scores for the KCPS. Regarding chewing performance, the KCPS revealed a significant difference for the total score for both T0 and T1 (*p* < 0.01) and T0 and T2 (*p* < 0.01) and between T1 and T2 (*p* < 0.05).

Regarding the modification of textures accepted by the child, the Friedman test revealed statistically significant differences in score variability with a *p* < 0.001 with a large effect size (Kendall’s W value 0.91), while the Wilcoxon signed-rank test of the IDDSI showed a significant difference was found immediately post-training, as well as between T0 and T2 with a *p* < 0.01. No differences were found between T1 and T2 (*p* = 0.10). Table 4 reports both the mean (SD) and median (IQR) scores for the IDDSI, as well as the frequency of IDDSI levels across different times of the intervention.

Additionally, while behavioral issues were not specifically investigated, it is notable that food refusal, anger, and crying gradually reduced during the treatment. By addressing both the sensory and motor aspects of the oral phase, the child improved their skills and was able to complete the tasks. Consistent sensory adaptation and chewing training are crucial for achieving better results and reducing problem behaviors. In the study by Ferrari and colleagues [27], an inverse relationship is observed between acceptance and exhibited behaviors. To maintain acceptance during a chewing intervention, it is beneficial to find the appropriate texture for the child’s current skill level, as foods with higher consistency tend to trigger problem behaviors. As oral sensory and motor skills improve, the occurrence of dysfunctional behaviors decreases. Maladaptive behavior, or problem behavior, generally includes oppositional behaviors such as tantrums, aggression, and disobedience, which interfere with optimal functioning and engagement with the environment. Although individuals with DS generally exhibit less maladaptive behavior compared to those with other developmental disorders, it is estimated that about one-third of individuals with DS have significant levels of maladaptive behavior [36]. Children with DS often display low-level aggressive behaviors and food-avoidant eating behaviors, such as fussiness and slowness in eating. Sensory processing disorders likely impact the maladaptive behavior profile in this population [37].

## 4. Discussion

This investigation provides preliminary evidence of the effectiveness of GIFT in children with Down syndrome (DS), concerning improvements in chewing performance (KCPS *p* < 001), texture acceptance and modification (IDDSI *p* < 0.001), both with a large effect size (Kendall’s W value > 0.8). A large effect size means that our research findings have a practical significance.

**Risk of Dysphagia.** One of the primary findings of our study is that children with DS demonstrated a low risk of dysphagia according to the PSPED score, with all participants scoring between 0 and 5, indicating a low risk [32]. The items that most contributed to the PSPED score were #4, 11, 13, and 3. Specifically, nearly 25% of the sample responded positively to item #4, which relates to respiratory and/or swallowing system alterations or malformations. The study by Jackson and colleagues [9] suggests that children with DS are more likely to have dysphagia due to anatomical and pulmonary characteristics. Consequently, parents often opt for safer foods to avoid dysphagia. However, childhood is a critical period for developing oral feeding skills, and feeding and swallowing behaviors can be influenced by both anatomical conditions and lack of experience. Additionally, 65% of the sample had gastrointestinal tract disorders (item #11). As described by previous studies [12,13], feeding problems and gastrointestinal disorders are common in individuals with DS, with abnormalities being either anatomical or functional. This study reports that gastroesophageal reflux disease (GERD) affects 47% of children with sleep apnea, constipation affects 19%, and obesity affects 32%. GERD is often pathological; however, pain is rarely expressed. Frequent GERD episodes can lead to difficulties in learning taste and oral hypersensitivity, which can alter taste buds. Moreover, 75% of the subjects responded positively to item #13, “Intake of food with a consistency not appropriate for age.” This finding aligns with the current literature [16], indicating that anatomical and physiological characteristics, such as disorders of neuromotor coordination and craniofacial anomalies, frequently interfere with the acquisition of effective sensorimotor skills, leading to potential feeding and swallowing problems. Families often favor blended or semi-solid foods, avoiding solid foods during the critical period of skill development [12]. Lastly, 40% of the investigated population had a history of cardiopathy (item #3). According to Lagan [6], congenital heart disease is frequently diagnosed in newborns with DS and often requires prompt surgical intervention. This results in numerous hospitalizations, specific drug therapies, and surgical procedures that can delay the development of oral sensory and motor feeding skills.

**Sensorial issues.** The IDDSI scale was used to assess the accepted consistencies of the participants, finding that 65% of the sample is at level 4, which involves the intake of very thick creamy foods administered by spoon. This result is in line with the literature, which suggests that the population with DS has a high propensity to present difficulties both in the introduction of new flavors and in the introduction of new textures to their usual diet [1]. In fact, sensory difficulties can have a negative impact on family routine, leading to different dietary needs from the rest of the family and dysfunctional mealtime behaviors. The study by Stein Duker et al. [38] reports that children with DS have difficulties in sensory processing, distinguishing two categories of sensory response: hyposensitivity and hypersensitivity to food stimuli presented in the oral cavity. Oral hypersensitivity leads to poor tolerance even in oral hygiene. The lack of oral hygiene in turn leads to the development of dental diseases that further hinder the development of chewing skills. After treatment, working in prerequisites and on the development of oral sensory and motor issues has led to a change in the accepted food textures. In fact, the entire study population has positively changed the accepted texture: 45% is at level 5 (minced); 30% at level 6 (chopped); and 25% at level 7 (normal). Thanks to the constant work of the caregivers, the entire sample at follow-up maintained the skills achieved at the end of the intensive treatment. The correct use of feeding tools, such as a glass and spoon, has improved tongue movements and reduced tongue thrust during food manipulation and swallowing. The correct alignment of the cup with mandibular support allows for greater stabilization of the jaw, reducing liquid loss from the oral cavity. Regulating the amount of solid and liquid food inserted into the oral cavity has allowed the child to develop greater awareness of hypo/hypersensitivity and oral motor skills of chewing and swallowing.

**Chewing abilities.** Our study revealed that children with DS exhibit chewing difficulties; in fact, a recent study by [27] suggests the importance of a more specific assessment of chewing skills that goes beyond examining only jaw movements but also includes functional lateral tongue movements, the initiation of rotary or vertical chewing, and the timing from food insertion into the oral cavity to swallowing.

The results of our study suggest that, at the first assessment, 80% of the sample is at level 4 of the KCPS scale (the child is unable to bite or chew). At the end of the rehabilitation program, 60% of the sample is at level 3 of the KCPS scale (they are able to bite and hold food between their teeth independently, but the subsequent steps of chewing are absent). At follow-up, 50% have maintained the skill and are at level 3. However, as suggested by the IDDSI results, the children are still able to achieve an increase in textures, reaching level 7 of the IDDSI scale (solid food diet), as the solid food is administered with guided chewing training. Through functional guided chewing training, the child trains to develop oral motor skills. In fact, a study by [39] suggests that the components involved in chewing only develop when children are exposed to foods of a higher consistency.

**The importance of parent involvement.** The GIFT treatment involves the presence of the caregiver so that they can observe, learn the rehabilitation techniques, and apply them in daily life. Following the GIFT intervention, there is a monitoring phase and subsequent follow-up after treatment completion. Optimal outcomes during follow-up are achieved through consistent home training. Once actively engaged in intensive training, parents must sustain their efforts. Caregivers should practice the learned techniques daily and apply them in various settings to reinforce and generalize newly acquired skills. Consistent with the existing literature, caregivers hold a crucial role in child feeding due to their firsthand experience with feeding behaviors, knowledge of food preferences and aversions, and understanding of communicative behaviors during meals [16]. The study by Stele and colleagues [40] reports that challenging feeding behaviors or feeding difficulties, commonly present in children with DS, can amplify perceived stress in caregivers. In particular, the study found that feeding difficulty is a significant stressor for caregivers of children with DS, especially during the transition to table food; however, as caregivers develop a variety of strategies for managing mealtime, their stress related to feeding difficulties decreases, and their sense of self-efficacy improves.

**Difference between GIFT in the ASD and DS.** As reported in the article by Cerchiari and colleagues [18], the GIFT program is effective for oral sensory motor disorders in children with ASD. This study demonstrates that GIFT is also effective in treating oral sensory and motor skills in children with DS and can be applied with similar objectives in both populations. Compared to its application in children with ASD, the GIFT program for children with DS also addresses the postural aspects due to the syndrome’s anatomical and physiological characteristics, such as generalized hypotonia, ligamentous laxity, and atlanto-occipital instability. Ensuring correct head-trunk-pelvis alignment is crucial for the treatment’s success. Additionally, during guided chewing training, proper mandibular support must be provided due to DS-related anatomical and physiological characteristics that negatively impact masticatory performance, including tongue thrust, mandibular instability, Class III malocclusion, hypotonia of the orofacial muscles, and oral breathing.

This study found that the children maintained the skills learned but did not improve their independent chewing ability during the home-based intervention period. Therefore, it can be hypothesized that achieving significant changes requires consistency and repetitiveness of functional exercises with food, intensive rehabilitation training with the GIFT protocol repeated over time, and daily exercises performed by parents at home. In the ASD population, age-appropriate feeding skills can often be achieved with a single intensive rehabilitation training session. The GIFT program for the ASD population spans two weeks with 30 feeding sessions, as the characteristics of the syndrome necessitate longer adaptation times to the setting. In contrast, the GIFT program for the DS population involves 15 feeding sessions over one week.

**Study Limitations.** Despite promising findings, it is important to acknowledge several limitations. The sample size is too small to generalize the results to the entire population with DS. Furthermore, no standardized tests or questionnaires were administered to assess mealtime behaviors, which were only described qualitatively. The follow-up results are strongly dependent on family collaboration in the home environment. Overall, these limitations suggest that the results should be interpreted with caution and that further research with a larger, more representative sample is needed to confirm the effectiveness of the proposed speech therapy treatment. Furthermore, it would be important, considering behavioral problems in this target population, to use specific assessment tools in stratifying the main characteristics to consider for compliance in rehabilitation and risks. In the end, we did not investigate the relationship between dysphagia and body mass index, neck circumference, as well as health comorbidity distribution and drugs used, while all these aspects can influence feeding and swallowing disorders and increase the risk of developing dysphagia. 

## 5. Conclusions

GIFT improves chewing ability, food acceptance, and increased food textures. It helps modulate behavioral problems at mealtime and involves families. In conclusion, it can be stated that GIFT seems to be an effective approach for children with DS, as it presents an individualized rehabilitation treatment that focuses on improving functions and specific limitations of children with DS.

## Figures and Tables

**Table 1:** Socio-demographic and clinical characteristics of the sample (total 20).

**Age Mean (SD)**	4.86 (2.43)
**Sex**	N (%)
Male	12 (60)
Female	8 (40)
**KCPS Level Distribution**	N (%)
Level 0	0 (0)
Level 1	0 (0)
Level 2	2 (12.5)
Level 3	2 (12.5)
Level 4	16 (75)
**IDDS mean (SD)**	4.40 (1.05)
**PS–PED mean (SD)**	2.80 (1.4)

**Table 2:** Frequency and % of each item of the PS–PED.

	PS–PED		
Item	Description	Yes *n* (%)	No *n* (%)
1	Neurological diagnosis	1 (5)	19 (95)
2	Epilepsy medications	0 (0)	20 (100)
3	Heart disease	8 (40)	12 (60)
4	Structural anomalies of the digestive and respiratory systems	5 (25)	15 (75)
5	Tracheal tube	0 (0)	20 (100)
6	Decreased alertness	0 (0)	20 (100)
7	Malnutrition and/or poor growth	0 (0)	20 (100)
8	Recurrent respiratory tract infections	6 (30)	16 (70)
9	Use of the suction machine/aspirator	0 (0)	20 (100)
10	Lack of head control and/or postural instability	0 (0)	20 (100)
11	Gastrointestinal diseases (gag reflex, vomiting, constipation, GERD)	13 (65)	7 (35)
12	Parenteral/enteral nutrition (nasogastric tube, gastrostomy tube, etc.)	0 (0)	20 (100)
13	Feeding with consistency and unsuitable food for the child’s development stage	15 (75)	5 (5)
14	Prolonged mealtime (over 50 min)	3 (15)	17 (85)

**Table 3:** Differences in scoring of the KCPS.

KCPS	N (%)			KCPS	T0	T1	KCPS	T0	T1	Sig
	T0	T1	T2							
**Level 0**	0 (0)	0 (0)	1 (5)	Mean (SD)	3.70 (0.66)	2.60 (0.75)	Median (IQR)	4.00 (4.00–4.00)	3.00 (2.00–3.00)	0.001 **
**Level 1**	0 (0)	2 (10)	4 (20)		**T1**	**T2**		**T1**	**T2**	
**Level 2**	2 (10)	5 (25)	3 (15)		2.60 (0.75)	2.35 (1.04)		3.00 (2.00–3.00)	3.00 (1.25–3.00)	0.025 *
**Level 3**	2 (10)	12 (60)	11(55)		**T0**	**T2**		**T0**	**T2**	
**Level 4**	16 (80)	1 (5)	1 (5)		3.70 (0.66)	2.35 (1.04)		4.00 (4.00–4.00)	3.00 (1.25–3.00)	0.001 **

* *p* < 0.05 ** *p* < 0.01.

**Table 4:** Differences in scoring of the IDDSI.

IDDSI	N (%)			IDDSI	T0	T1	IDDSI	T0	T1	Sig
	T0	T1	T2							
Level 7	1 (5)	5 (25)	8 (40)	Mean (SD)	4.40 (1.05)	5.80 (0.83)	Median (IQR)	4.00 (4.00–4.00)	6.00 (5.00–6.00)	0.001 **
Level 5–6	5 (25)	15 (75)	12 (60)		**T1**	**T2**		**T1**	**T2**	
Level 4–3	13 (65)	0 (0)	0 (0)		5.80 (0.83)	6.00 (0.91)		6.00 (5.00–6.00)	6.00 (5.00–6.00)	0.10
Level 2–1	1 (5)	0 (0)	0 (0)		**T0**	**T2**		**T0**	**T2**	
Level 0	0 (0)	0 (0)	0 (0)		4.40 (1.05)	6.00 (0.91)		4.00 (4.00–4.00)	6.00 (5.00–6.00)	0.001 **

** *p* < 0.01.

## Data Availability

The data presented in this study are available on request from the corresponding author. The data are not publicly available due to restrictions, e.g., privacy or ethical.
